# Primary Care Quality Improvement Metrics and National Committee on Quality Assurance Medical Home Recognition for Children With Medical Complexity

**DOI:** 10.1097/pq9.0000000000000231

**Published:** 2019-11-26

**Authors:** Jennifer Lail, Elise Fields, Alyssa Paolella, Pamela J. Schoettker

**Affiliations:** From the * James M. Anderson Center for Health Systems Excellence, Cincinnati Children’s Hospital Medical Center, Cincinnati, Ohio; † Department of Pediatrics, University of Cincinnati College of Medicine, Cincinnati, Ohio; ‡ General and Community Pediatrics, Cincinnati Children’s Hospital Medical Center, Cincinnati, Ohio.

## Abstract

The Complex Care Center at Cincinnati Children’s Hospital Medical Center developed and implemented a set of evidence-based clinical process measures of immunization delivery, preventive and chronic condition laboratory screening, and behavioral health medication surveillance for use in the primary care setting.

**Methods:**

Improvement activities focused on 4 measures across 4 domains mandated by the National Committee on Quality Assurance for patient-centered medical home recognition. Interventions were implemented in phases from January 2017 to October 2018. The goal was the improvement in immunizations, preventive lead screening, vitamin D testing in chronic conditions, and behavioral health medication surveillance. Preventative lipid screening in the entire population and thyroid-stimulating hormone levels in patients with Down syndrome were also measured.

**Results:**

The offering and the completion of an immunization bundle increased from a mean of 61.0% to a mean of 83.7% of patients. Eligible patients with documented lead surveillance increased from 61.2% to 96.5%. Patients with documented 25-hydroxy vitamin D levels increased from 72.2% to 87.8%. The percentage of patients metabolically monitored while taking an atypical antipsychotic continued at 92.0%.

**Conclusions:**

Using quality improvement education, data measurement/feedback, electronic medical record optimization/decision support, and care gap identification with planned care, the Complex Care Center demonstrated improved reliability in needed immunization delivery and laboratory screenings for a heterogeneous primary care population of children with medical complexity. As the numbers of children with medical complexity rise, so does the importance of reliable processes and relevant measures for quality in their unique care delivery systems.

## INTRODUCTION

The numbers of children with medical complexity and their impact on the US health system are increasing.^1,2^ These children have heterogeneous diagnoses, multiple organ system involvement, medical fragility, functional impairments, and often have a dependence on technology for survival and activities of daily living. Beyond frequent hospitalizations, these children require intense family and professional care coordination for their primary and specialty medical care, therapies, multiple medications, adaptive educational services, and medical equipment. They may also require home care services. Optimal care delivery systems for children with medical complexity are evolving, with primary care medical home, co-management, and episode-based care models tested for efficacy.^3^

Fewer well-child visits in this population is associated with more hospitalizations,^4^ but well-child care, vaccines, screenings, and condition-specific surveillance recommended by the American Academy of Pediatrics, are often missed.^4^ Given the complexity of their daily care, these children may miss well-child care due to insurance barriers, lack of access to a primary care medical home, competing specialty care and therapy appointments, recurrent medical instability and social factors, such as lack of adaptive transportation, parental work absence and/or difficulty with organization and planning.

American Academy of Pediatrics Bright Futures’ standards for preventive care, developmental and mental health screening, disease prevention, health promotion, nutrition, growth, and development are directed toward the population norms of healthy children.^5^ These recommendations may require adaptation for children with medical complexity, who may have vaccine contraindications, neurologic impairment, functional impairments, or technology-dependence for function and survival. Building and measuring highly reliable primary care processes applicable to this population are important.

Given their severe and infrequent conditions, identifying population-level quality metrics relevant to the children with complex conditions, their clinicians and families, is challenging.^6^ The Complex Care Center (CCC) at Cincinnati Children’s Hospital Medical Center (CCHMC), using the patient-centered medical home model and an electronic medical record (EMR)-based population registry, documented prior improvement in their provision of reliable well-child care, vaccination rates for children 0–7 year of age, and electronic pre-visit planning processes for population management.^7^ Standards from the National Committee on Quality Assurance (NCQA) include core competencies focused on care coordination, care transition, and self (family) management that are key to providing complex, chronic care. While pursuing NCQA patient-centered medical home recognition,^8^ the CCC continued quality improvement (QI) interventions, implementing evidence-based clinical process measures of vaccine delivery, preventive and chronic condition laboratory screenings, and behavioral health for their population. We report here on those interventions and measures.

## METHODS

### Setting

The CCC functions as an outpatient medical home, within the Division of General and Community Pediatrics at CCHMC, for 449 children with medical complexity ranging in age from birth to 23 years and for 78 less complex patients and siblings. Patients are stratified by medical complexity using the electronic Pediatric Medical Complexity Algorithm (PMCA).^9^ Tier-3 patients are considered most complex. Patients eligible for services at the CCC meet at least 1 of 3 criteria: they are dependent on some technology to stay alive,^10^ have ≥3 chronic medical conditions, or need access to ≥3 major-organ-system specialists. CCC patients see an average of 7 specialists. While some patients have multiple insurers, 87.0% have some form of Medicaid.

The CCC holds 9 clinical sessions per week at 2 hospital campuses. Social work, care management, and nutrition support are available at each session; a medical provider is on call 24 hours a day. The CCC co-manages care with patients and their families, specialists, therapists, home health, medical equipment suppliers, and community partners, such as schools and camps.

### Study Population and Measures

In January 2017, anticipating participation in the NCQA Patient-Centered Medical Home Recognition Program,^8^ the physician leader conducted a literature review of the evidence base for clinical process measures meaningful to the population of children with medical complexity. To meet NCQA requirements,^11^ we gathered data on 6 clinical quality measures across the 4 domains, setting goals to improve 4 and monitor 2 more. We developed operational definitions that included rationales, population definitions with inclusions and exclusions, data collection plans, and methods of calculation, analysis, and data reporting. Information Services provided support for data collection within the EMR and the James M. Anderson Center for Health Systems Excellence provided reporting and data analytic support.

The study population consisted of all CCC patients who were alive, had completed a visit in the past 36 months, and had not transitioned or transferred their care. Some measures applied to subpopulations of patients (defined below). Goals were chosen based on a 12-month retrospective data average for 2016.

The 4 main measures sought to (1) ensure completion of vaccine administration and (2) lead screenings; (3) improve measurement of vitamin D levels; and (4) improve receipt of recommended laboratory and clinical surveillance for patients taking atypical antipsychotics.^12^ These evidence-based measures were selected because they were clinically relevant for patients, applicable across the diverse diagnoses in complex care, and aimed at preventing complications. For example, known risks of vitamin D insufficiency in children with medical complexity exist.^13^ Completion of lipid screenings for patients 9–11 years old was monitored as recommended by the Bright Futures initiative,^14^ and thyroid-stimulating hormone levels followed as part of the national guidelines for care in patients with Down syndrome.^15^ Finally, as part of ongoing quality monitoring, the team followed their reliable rates of well-child care completion and vaccine completion for children 0–7 years of age.

For the immunization domain, we developed a visit-based measure to improve on the percent of patients 11–13 years of age advised to receive tetanus, diphtheria, acellular pertussis, meningococcal, and human papillomavirus vaccines (goal 60.0%). Data from the Centers for Disease Control and Prevention showed HPV vaccine coverage lagged behind other vaccines in this adolescent bundle^16^ and may require repeated discussion to promote acceptance.^17^ Accordingly, refused vaccines were documented, counted as successful attempts to advise, and recommended at subsequent visits until all vaccines were received. To calculate a monthly data point, we established the numerator as the number of patients 11–13 years of age who completed a visit and received (or were advised and declined) the vaccine bundle. The denominator was the number of all patients 11–13 years of age who completed a visit.

For preventive care, we measured the percent of patients aged 15 months to 72 months, with 1 documented lead level in the EMR (goal of 90.0%). The numerator was the number of patients 15–72 months with a documented lead level, and the denominator was the total number of patients between 15 and 72 months of age.

In chronic care, we aimed to have 80.0% of PMCA tier-3 patients with 1 25-hydroxyvitamin D result documented in the EMR within the past 15 months. The numerator for the measure was the number of PMCA tier-3 patients with a vitamin D level documented within the past 15 months. The denominator was all PMCA tier-3 patients in complex care.

The behavioral health measure selected for improvement was metabolic monitoring of the subset of children taking atypical antipsychotic medications (Olanzapine, Clozapine, Quetiapine, Risperidone, Ziprasidone, Aripiprazole, Paliperidone, Cariprazine, or Lurasidone), as per national recommendations.^12,18,19^ The goal was to increase the percentage of those patients with 1 documented body mass index, weight, glucose, and lipid screening in the EMR in the past 15 months to 93.0% using segmental lengths^20^ for height measurement/body mass index calculation when standing height was not possible. The numerator was the number of patients prescribed those atypical antipsychotic medications and with all components of the measurement bundle documented in the EMR in the past 15 months. The denominator was all patients prescribed these specific medications.

Per NCQA standards, 1 monitoring measure from both the preventive and chronic care domains was chosen: (1) the percentage of all CCC patients 11–12 years of age with a documented cholesterol and lipid screening received when they were 9–11 years of age, and (2) measurement and documentation of thyroid-stimulating hormone level within 15 months for the subpopulation of children with Down syndrome. We set no specific improvement goals. The EMR build of electronic best-practice alerts included these monitoring measures.

### Measure Implementation

Four overlapping groups of interventions were applied to the measures across 4 phases, from January 2017 to October 2018 (Table 1). Usage of QI education, data feedback, pre-visit planning, and clinic huddles for care gap management crossed these time phases. Providers and staff selected, developed, and used evidence-based measures for improvement. Next, a clinic pediatrician conducted monthly QI work sessions to teach staff and providers QI fundamentals and how to map processes, review and analyze data, provide decision support around measures, and plan process interventions using plan-do-study-act cycles.^21^ Measures were developed and validated by chart review and baseline data collected to inform ongoing data collection within the EMR.

**Table 1.**
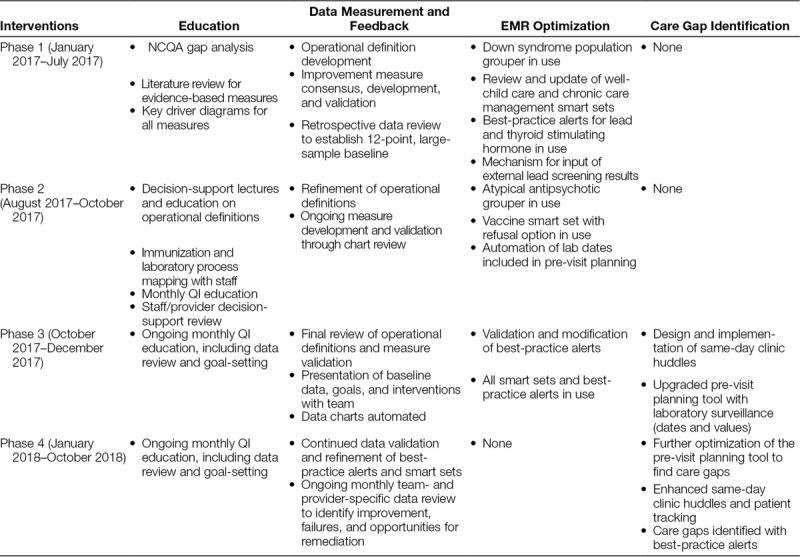
Interventions and Phases

A physician and a business leader used serial tests of change^21^ to develop electronic alerts, and tested and validated draft versions with provider feedback before final deployment in the EMR. Development and implementation of best-practice alerts and standardized order sets (“smart sets”) in the EMR provided electronic decision support to identify care gaps during patient interactions and streamlined order completion.

Along with EMR optimization, these interventions added cumulative support for the identification of care gaps. For example, the team used electronic templates for chart review of interim care, hospitalizations, and procedures during pre-visit planning for all well-child and chronic care management visits (by either registered nurse care managers or clinic providers) to identify needed care and the CCC team member with the expertise to address it.^7^ In brief pre-clinic huddles, staff led tests of change to communicate noted care gaps optimally, ultimately choosing a clinic wallboard to document needs and the responsible team member. This communication allowed social workers, dieticians, and care managers to predict their involvement within the face-to-face visit. At the visit itself, EMR prompts and order sets facilitated the completion of missed care.

Each month, in practice-wide QI meetings, the physician leader presented both raw data on missed opportunities and data charts showing changes over time. Provider-specific data were also available. This feedback identified opportunities to improve processes and allowed team members to identify and close gaps in care.

### Data Collection and Analysis

We developed operational definitions and identified subpopulations. Twelve averaged monthly data points from January 1, 2016 to December 31, 2016, were used to determine the baseline for each measure. The improvement period was January 2017 through October 2018. We obtained data for each measure from the EMR. Annotated charts^22,23^ were used to track changes in visit-based vaccine administration, atypical antipsychotic surveillance, and thyroid-stimulating hormone measures over time. Data for lead, vitamin D, and lipid screenings were auto-correlated (comparing each patient against their prior values and performance). To determine if interventions had a significant effect on the auto-correlated data, we performed an interrupted time-series analysis for these 3 measures.^24^ A statistically significant change in the slope (<0.05) indicated a significant change in trend after the intervention, compared with before the intervention.^21^ The autoregressive models of the 3 measures used the maximum likelihood method and were built using SAS statistical software.^25^ We used the Durbin-Watson test for autocorrelation in the final regression models.

### Human Subjects Protection

The Institutional Review Board at CCHMC judged this project to be QI and not human subjects research. Therefore, review and approval were not required.

## RESULTS

### Clinical QI Measures

**Fig. 1.**
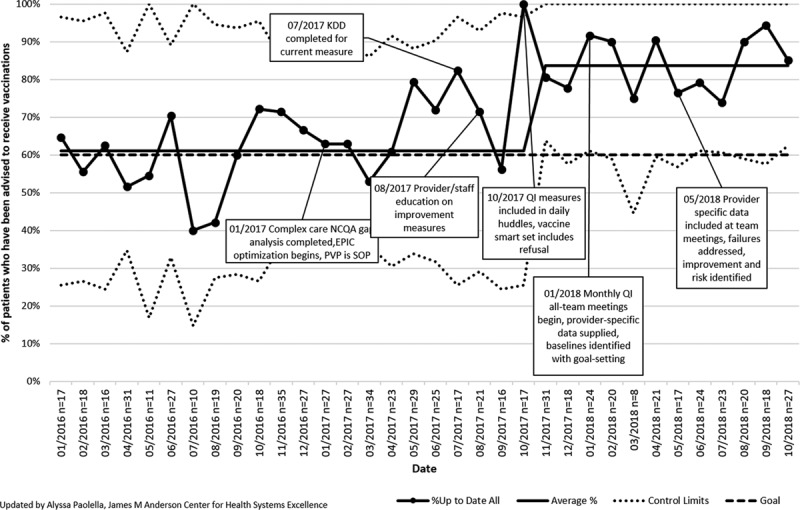
Percent of CCC active subpopulation patients 11–13 years old who were advised to receive tetanus, diptheria, acellular pertussis, and meningococcal, and human papillomavirus vaccines for which they were eligible at the time of the visit. KDD, key driver diagram; PVP, pre-visit planning; SOP, standard operating procedure.

By October 2018, there was a documented improvement in 3 of the 4 clinical QI measures and unanticipated high reliability^26^ in the fourth measure. The mean percentage of patients 11–13 years of age who received the vaccine bundle, or to whom the vaccine was documented to be offered and declined (n = ~22 patients/mo) increased from a baseline of 61.0% (95% CI, 59.7%–62.4%) to a mean of 83.7% (95% CI, 82.5%–85.0%; Fig. 1). The average percentage of documented vaccine refusals increased by 9.9% from October 2017 forward. The average percentage of patients receiving the vaccines did not increase.

**Fig. 2.**
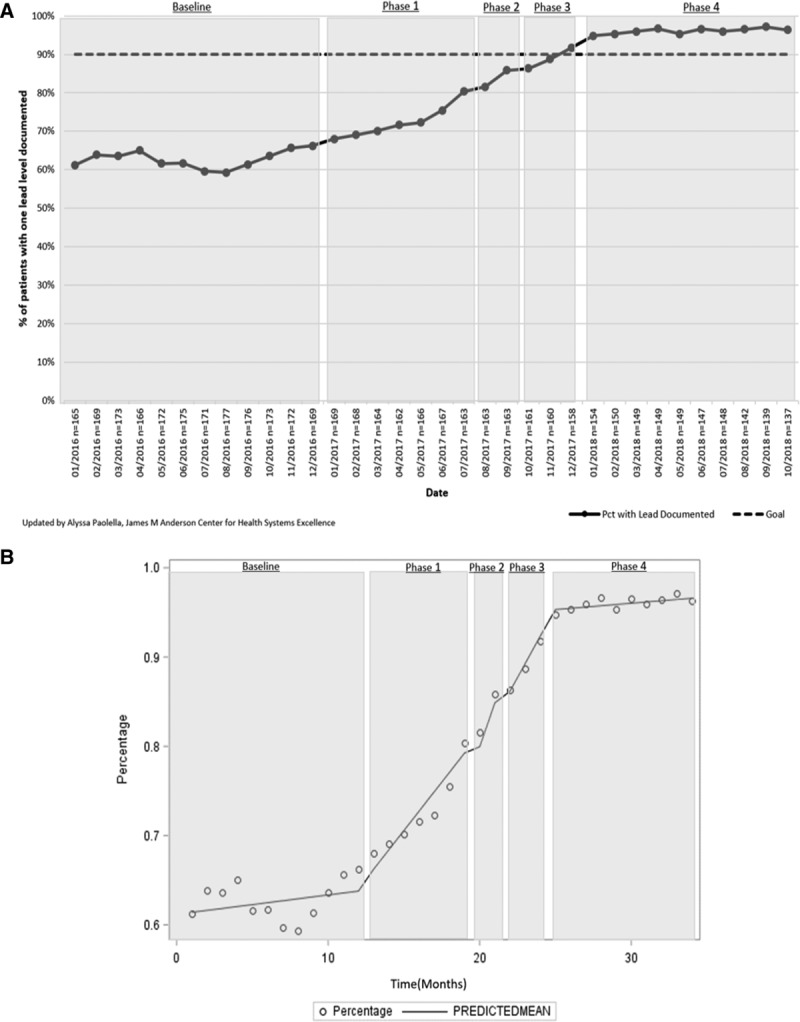
Percent of CCC active subpopulation patients 12 months to 72 months of age with 1 documented lead level result in the EMR. Pct, percent of patients. See Table 1 for interventions in each phase.

**Fig. 3.**
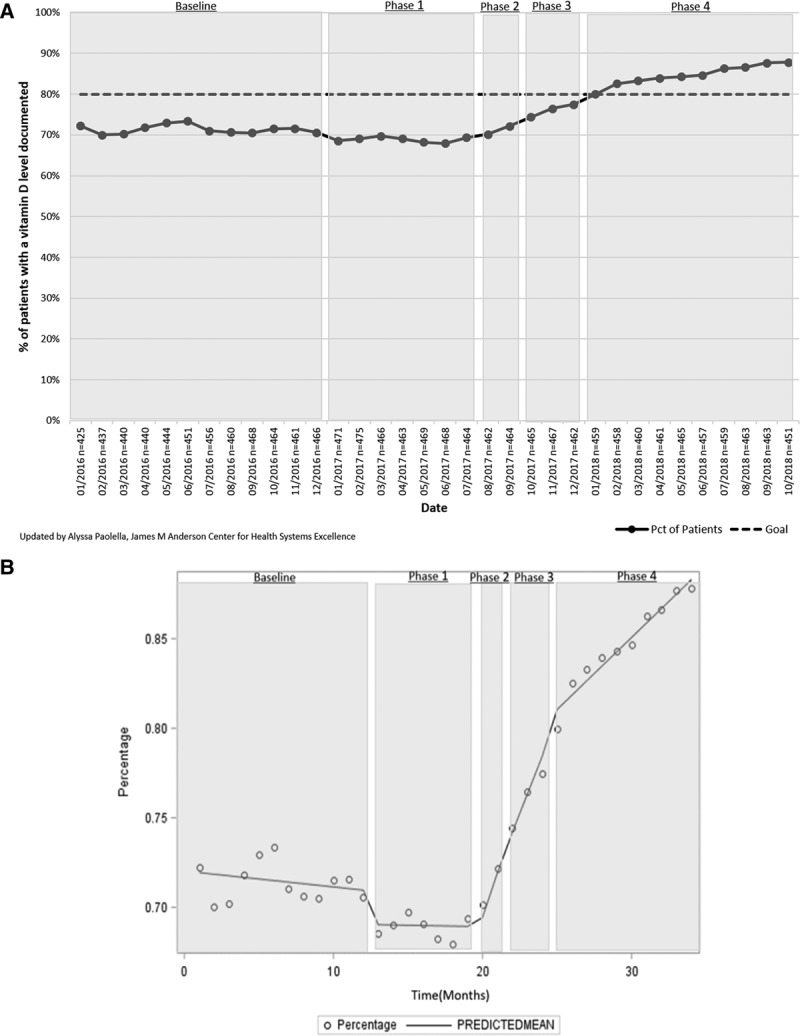
Percent of Complex Care Center active subpopulation patients with 1 vitamin D level documented in the EMR in the past 15 months. Pct, percent of patients. See Table 1 for interventions in each phase.

**Fig. 4.**
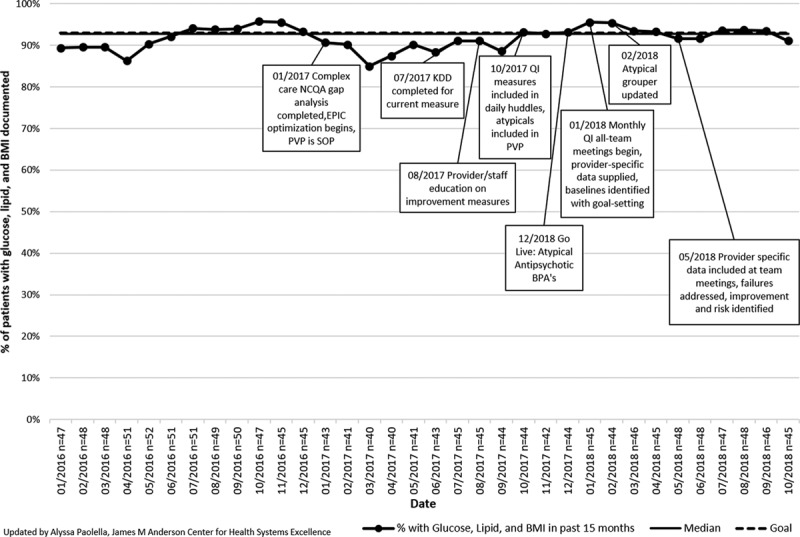
Percent of Complex Care Center active subpopulation patients currently on atypical antipsychotic medications with a glucose screen, lipid screen, and body mass index documented in the EMR in the past 15 months. BPA, best-practice alert; BMI, body mass index; KDD, key driver diagram; PVP, pre-visit planning; SOP, standard operating procedure.

For both the lead and vitamin D measures, the interrupted time-series analyses showed a positive slope trend and significant changes in slope in at least 1 intervention phase for each measure. Patients with 1 documented lead level result in their chart (n= ~161 patients/mo) increased from 61.2% (95% CI, 56.6%–66.1%) to 96.5% (95% CI, 93.8%–100.0%), exceeding the 90% goal (Fig. 2A). There was a statistically significant change in slope after intervention phase 1 (Fig. 2B). Patients with 1 vitamin D level documented in their chart (n= ~459 patients/mo) increased from 72.2% (95% CI, 69.7%–74.2%) to 87.8% (95% CI, 86.8%–90.1%), exceeding the 80.0% goal (Fig. 3A). There was a significant change in slope after intervention phase 2 (Fig. 3B). Both the lead and vitamin D measures had no autocorrelation in their final regression models, suggesting that the observed improvement in these measures was a result of the interventions. The percentage of monitored patients taking an atypical antipsychotic (n= ~46 patients/mo) continued at 92.0% (95% CI, 91.7%–92.2%; Fig. 4).

### Clinical Quality Monitoring Measures

**Fig. 5.**
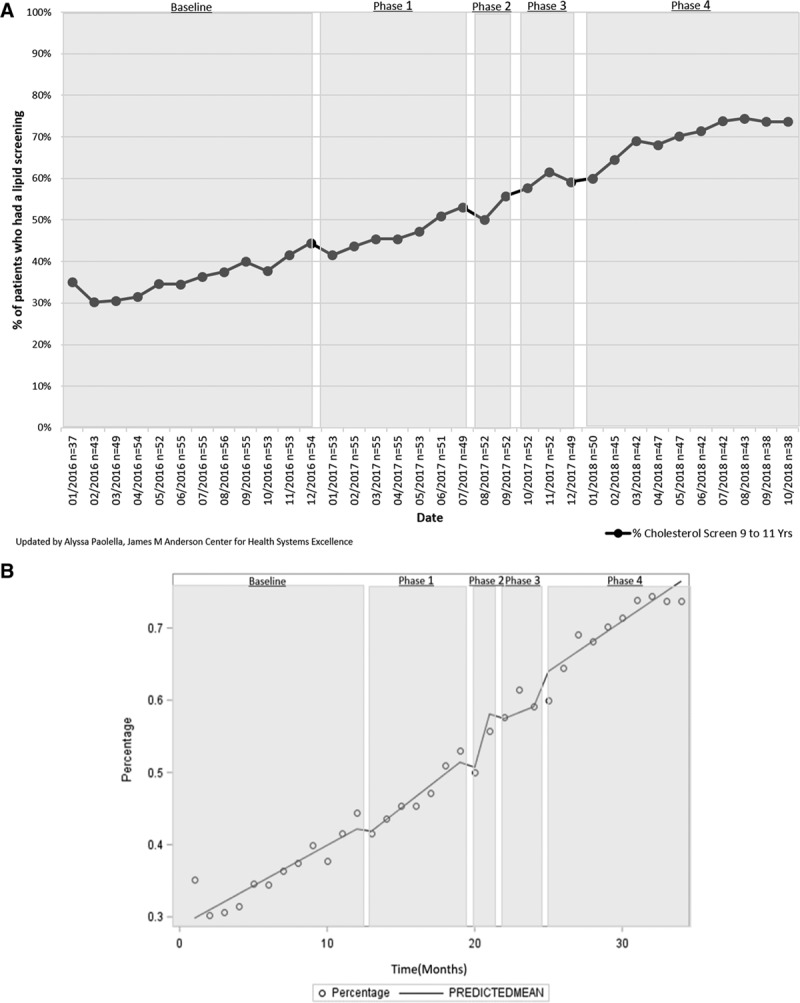
Percent of Complex Care Center active subpopulation patients 11–12 years old with a cholesterol/lipid screening between 9 and 11 years of age. See Table 1 for interventions in each phase.

There was a documented improvement in the 2 clinical quality monitoring measures. Thyroid-stimulating hormone levels in patients with Down syndrome (n= ~30 patients/mo) increased from 93.6% (95% CI, 93.2%–94.0%) at baseline to 97.6% (95% CI, 97.4%–97.9%) in October 2018. Patients who had a cholesterol/lipid screening (n = ~49 patients/mo) increased from 35.1% (95% CI, 24.7%–34.9%) to 73.6% (95% CI, 71.5%–80.2%; Fig. 5A). There was a significant change in slope for the lipid screening measure after intervention phase 2. There was no autocorrelation in the final regression model (Fig. 5B).

Adding the new measures created for NCQA patient-centered medical home recognition did not affect previous improvement measures related to completed well-child care and proper vaccine bundles for patients 0–7 years of age. Completed well-child visits remained unchanged at 90.0%, and delivery of the vaccine bundle for 0- to 7-year olds remained at 95.0%.

## DISCUSSION

By implementing education, EMR optimization with clinical risk groupers, best-practice alerts, order sets, and monthly data feedback, the clinicians and staff of the CCC improved vaccine delivery, lead screening, vitamin D measurement, thyroid-stimulating hormone measurement, and lipid screening, while maintaining high reliability in metabolic surveillance for patients taking atypical antipsychotics. Monitoring measures also improved. These improvements enhanced prior QI work,^7^ sustaining completion of the vaccine bundle for children 0–7 years old, and well-child care for the CCC population. While disruptions of care and appointments by hospitalizations and illness can be barriers to continued care, the frequent need for laboratory testing across these children’s multi-specialty care affords more opportunities for samples to be collected.

Lack of immunizations increase infectious risk in this fragile population, and lead exposure risk may be missed. Children with medical complexity may have immobility, non-weight-bearing, and anti-epileptic drug use as risk factors for vitamin D insufficiency and pathologic fractures, and those taking atypical antipsychotics have known risk for obesity and lipid derangements. Before this project, the clinicians and staff of the CCC were unaware of their performance on many measures, and baseline vaccination data showed variation. For example, we suspected poorer metabolic monitoring than was the case. As a result, execution on some of the measures was high at baseline and did not improve appreciably.

While more testing is needed across clinical settings, ongoing monthly provider and staff QI education, monthly feedback of performance data, and daily electronic decision support appeared as valuable approaches for improvement. Daily opportunities for care gap closure were identified in the electronic pre-visit planning template and highlighted in the pre-clinic huddle process. Staff were empowered to begin discussing vaccines with families and to enter, if appropriate, the code documenting vaccine contraindication or refusal by family. A review of missed opportunities for immunization showed that acute care, same-day care, and preoperative visits were associated with fewer vaccine care-gap closures, possibly because the child was ill at that visit, or prior care planning for same-day visits was less reliable. Information on vaccine completion versus refusal showed higher refusal rates for human papillomavirus vaccines than the meningococcal and tetanus, diphtheria, and pertussis vaccines and provided retrievable data on the population for whom the HPV vaccine was recommended but declined. With this information, the team plans future QI work on human papillomavirus vaccine completion.

The measure selection process and tools built deliberately involved measures that were cross-cutting across large and small populations (vaccines and lead) and were meaningful across multiple specialties caring for these children. These tools can be deployed across specialties within the EMR. For example, the smart set builds for thyroid-stimulating hormone and vitamin D measurements were reusable by Endocrinology, Nephrology, and other divisions contributing to the child’s care. The antipsychotic medication grouper was useful for Psychiatry and Endocrinology. The development and implementation of best-practice alerts and standardized order sets in the EMR heightened attention to improvement, provided electronic decision support, and facilitated order entry to close care gaps, as did the identification of children taking antipsychotic medications in the pre-visit planning template.

We displayed a verification of improvement across intervention phases 1 and 2 for the 3 auto-correlated measures. Because we bundled our interventions into phases and some of the interventions were cumulative, it is difficult to identify the specific interventions that contributed to the statistically significant differences in slope without further analysis. The best-practice alerts may explain the large increase in the percent of patients with a lipid screen between 9 and 11 years of age, although we applied no active interventions there. Monthly data feedback and analysis at the team and provider levels highlighted missed opportunities for remediation and encouraged the team’s progress.

While we did not measure the impact of the initiative on providers and staff, they were generally responsive to applying these streamlined process changes and asked for feedback on progress. Staff anticipated and participated in plan-do-study-act cycles and the monthly interactive QI education sessions with data review. Some staff chose to lead in their area of interest (eg, vaccine discussion and refusal). Provider/staff turnover or absence did require ongoing education about the initiative, processes, and measurement. Our highly dedicated staff helped new members to learn tools and processes.

Chen et al^27^ described the development of 35 primary care quality measures for complex pediatric patients based on a patient-centered medical home framework of accessible, continuous, family-centered, coordinated, and culturally effective care. Two of those measures focused on chronic care management plans and nutrition. That expert panel’s measures align with the structural standards required for NCQA recognition and informed the development of processes in the CCC. However, the evolving population of children with medical complexity^28,29^ will require the application of those foundational measures and measures specific to the unique needs of their rarer and heterogeneous conditions. Our results show that measurable and meaningful process improvement to find and close care gaps in the primary care of children with medical complexity is achievable. These efforts also contributed to attaining patient-centered medical home recognition from the NCQA in October 2018.

The limitations of this study may reduce its generalizability. The CCC operates in a large, free-standing children’s hospital and is committed to a complex care primary care model, potentially inaccessible to families at other locations. Other health systems may not have a similar capacity to optimize electronic support within the EMR. The fact that the majority of the CCC patients receive all of their care within the CCHMC hospital system, and it uses an EMR, may have facilitated the completion and tracking of labs and vaccines.

In conclusion, more work is needed to determine the efficacy and fiscal viability of comparative models of care for children with medical complexity, including the patient-centered medical home primary care model, the Chronic Care Model,^30–34^ and others. Identifying measures for the care of this unique population will be critical to assessing which models of care work best and what supports they require. As diverse models of care emerge, continuous QI efforts and measurement will add to the growing understanding of effective, safe, and affordable care for children with medical complexity. Ultimately, measurement of the child’s clinical and functional outcomes, and effective family engagement and support mechanisms, will be critical for this emerging population.

## ACKNOWLEDGMENTS

The authors thank providers and staff in the CCC, analysts Kate Rich, Allison Glance, and Pavan Chundi in the James M. Anderson Center for Health Systems Excellence, and Jude Hayden in the Department of Information Services.

## DISCLOSURE

The authors have no financial interest to declare in relation to the content of this article.
